# Efficient photocatalytic degradation of petroleum oil spills in seawater using a metal-organic framework (MOF)

**DOI:** 10.1038/s41598-022-26295-8

**Published:** 2022-12-23

**Authors:** M. S. Showman, Asmaa M. Abd El-Aziz, Rana Yahya

**Affiliations:** 1 Fabrication Technology Research Department, Advanced Technology and New Materials Research Institute, City of Scientific Research and Technological Applications, Alexandria, Egypt; 2 Department of Chemistry, College of Science, University of Jeddah, Jeddah, Saudi Arabia

**Keywords:** Environmental sciences, Materials science

## Abstract

Photocatalysis is a green approach that has appeared to be a viable option for the degradation of a variety of organic contaminants. This work outlines the process of preparing the titanium-based metal-organic framework (MIL-125) photocatalysts using a simple solvothermal method. Structural, morphological, and optical analysis of samples (MT18 and MT48) was carried out by XRD, FT-IR, Raman, SEM, TGA, BET, and UV–Vis. Results indicated that the sample prepared at 150 °C and reaction time of 48 h (MT48) has a low crystal size of 7 nm with an optical band gap of 3.2 eV and a surface area of 301 m^2^ g^−1^. Under UV–visible light irradiation, the as-prepared MOFs proved to upgrade photocatalytic activity in degrading crude oil spills in saltwater. Effects of catalyst dosage and exposure time on the degradation of an oil spill in seawater were studied and analyzed using UV–Vis spectrophotometry and gas chromatography (GC–MS) which emphasized that the use of 250 ppm of MT48 photocatalyst under UV–Vis irradiation can degrade about 99% of oil spills in water after 2 h of exposure. The study's data revealed that MIL-125 could be used to photocatalyzed the cleanup of crude oil spills.

## Introduction

Marine oil spills typically relate to human activities that result in the release of liquid petroleum hydrocarbons into the ocean or coastal areas. They entail the release of crude oil from ships, offshore platforms, drilling rigs, and wells. Spills of crude oil can cause enormous economic losses and damage to ecological systems, public health, and communities^1^. Crude oil can be used not only as fuel and gasoline but also as a raw material for industrial products, such as fertilizers, pesticides, and plastics^2^. Dissolution, emulsification, absorption, mixing, evaporation, biodegradation, photodegradation, and chemical reactions all contribute to the degradation and dispersion of aquatic discharged oil^3^.

Photocatalytic oxidation is a method of converting oil into carbon dioxide, water, and salts using light as a driving energy source. Catalyzed advanced oxidation processes (AOPs) in aqueous media generate hydroxyl radicals, which are highly reactive with nonselective chemicals and have a high oxidation potential (E_o_ = 2.8 V) to destroy petroleum hydrocarbon molecules, allowing them to react with a wide range of oil pollutants^4^.

Metal-organic frameworks (MOFs) have attracted the interest of researchers because to their unusually large specific surface areas and open, interconnected microporous architectures, a high proportion of transition metals, and the capacity to be customized and modified after synthesis, and their ability to exhibit semiconductor-like behavior^5,6^. To date, more than 20,000 MOFs have been designed and synthesized. Most of those MOFs' applications, however, are hampered by their unstable nature. Aiming at realizing a more stable MOF, extensive efforts have been made by researchers around the world. Among the various semiconductors, TiO_2_ is the first example used for photocatalytic hydrogen production due to its light sensitive Ti ions. Superior to TiO_2_, MIL-125 not only possess Ti-oxo clusters or Ti-oxo chains/sheets, but also have light harvested ligands, endowing them with promising photocatalytic activity. The adjustable structures of MIL-125, in particular, allow them to efficiently utilise solar light beyond the ultraviolet region (which accounts for only 4% of the total)^7–9^. The stability properties of MOFs mainly contain four factors, such as chemical stability, photostability, mechanical stability, and thermal stability^10,11^.

In the photocatalysis mechanism, photogenerated electrons and holes are separated and used in the same way as they are in traditional semiconductors^12,13^. When the photon energy is greater than or equal to the MIL-125 bandgap, the MIL-125 can be stimulated by light to generate photo-generated electrons and holes^14,15^. Electrons are stimulated from the highest occupied molecular orbital (HOMO) of the organic linker to its lowest unoccupied molecular orbital (LUMO). The photoexcited electrons are then passed to the MOF nodes via a linker-cluster charge transfer mechanism (LCCT), which activates the metal nodes and allows them to participate in the photooxidation reduction reaction^16,17^. As a result, boosting photocatalytic activity requires improvements in the quantity of generated electron–hole pairs, separation efficiency, and carrier utilization efficiency.

Direct synthesis of MIL-125 using traditional hydrothermal/solvothermal procedures is still the most widely utilized approach^18^. Solvents have a vital role in the crystallinity of finished goods, and the ideal solvent combination is often discovered by trial-and-error. Another important aspect that influences the shape of MOFs formed is the concentration of reactants^19^. Herein, we present a simple and efficient scheme to fabricate catalytic MOF by a solvothermal process. Furthermore, this study presents a novel and highly efficient photocatalytic degradation of crude oil spill using MIL-125.

## Materials and methods

### Materials

Terephthalic acid (H_2_BDC), HPLC methanol (MeOH), and anhydrous di-methyl formamide (DMF) were purchased from Fischer Scientific, UK. Titanium isopropoxide (TTIP) was produced in Sigma Aldrich, USA. Saline Water (TDS = 27,800 ppm) was taken directly from the Mediterranean Sea, Alexandria, and crude oil from the western desert, Egypt with the main characteristics shown in Table S1.

### Methods

#### Preparation of MIL-125

**Figure 1:**
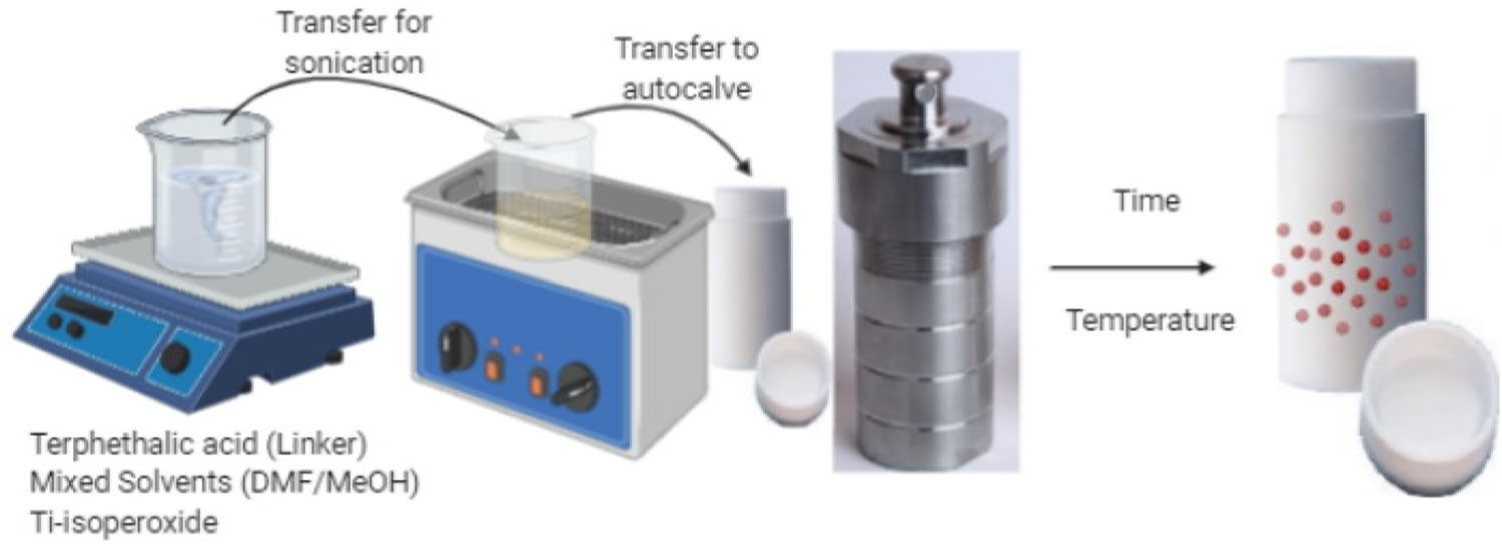
Detailed preparation methods of the solvothermal preparation of Ti-MOFs.

The preparation method is shown in Fig. 1; in which 6.0 mmol of terephthalic acid (H_2_BDC) was first dissolved into a mixed solvent of methanol (MeOH) and anhydrous dimethyl formamide (DMF) with a ratio 1:9. The solution was then treated with 1.9 mmol of titanium isopropoxide (TTIP) Ti(OCH(CH_3_)_2_)_4_ for 1 h while stirring. The mixture was then sonicated for 25 min and transferred into a stainless-steel autoclave system (volume: 150 mL), reacting at 150 °C for different reaction times (18, 48) h^9,20–22^. To eliminate other unreacted organic species in the mixture, the yellow solid result was washed twice with a mixed solvent of (DMF and methanol). Afterward, the solid product was isolated by centrifugation. Finally, the white powder was acquired after vacuum drying it overnight in an oven at 80 °C^23^. The prepared MIL-125 samples were coded (MT18, MT48).

#### Characterization of the prepared MOFs

##### Fourier transform—infrared (FT-IR)

The functional groups of MIL-125 samples were investigated using a Fourier transform—IR spectrophotometer (FTIR-8400S, Shimadzu) with a resolution of 2 cm^−1^. The powder samples were tested in the (4000–350) cm^−1^ wavenumber range.

##### Scanning electron microscope (SEM)

Scanning electron microscopy (SEM) (JEOL JSM 6360LA, Japan) was used to investigate the microstructure and surface morphology of all prepared samples (MT18, MT48). Before SEM imaging, the powders were sputter-coated with gold using a sputter-coating unit.

##### Raman analysis

A SENTERRA Raman Microscope with laser selectors (532, 633, and 785) nm was used to collect Raman spectra; the wavelength and power of the laser employed as an excitation source were 514.5 nm and 20 mW, respectively. The incident light was focused on the prepared samples and the radiation scattered by the sample was collected using a 50 objective (back-scattered configuration). A single spectrum required 360 s to acquire.

##### x-ray diffraction (XRD)

X-ray diffraction scans of samples were obtained using (X-ray 7000 Shimadzu-Japan) at room temperature. The degree of crystallinity was determined by applying Bragg's low where 2θ is in the range of 5°–80°, with the X-ray source being a Cu target produced at 30 kV and 30 mA and a scan speed of 4° per minute. The crystallite size is determined from the broadening of corresponding X-ray spectral peaks by the Scherrer formula^24^:

$$\mathrm{D}=\mathrm{K \lambda }/(\mathrm{\beta cos \theta })$$

$$\upbeta =[\mathrm{FWHM}\times (\pi/180)]$$

where D is the crystallite size (dimension), λ is the wavelength of the X-ray radiation (Cu Kaα = 0.15418 nm), K is a constant (shape factor) usually taken as 0.89, θ is the angle between the incident and reflected rays and β is the line width at half-maximum height.

##### Thermogravimetric analysis (TGA)

Thermogravimetric analysis is a sort of testing that is done on samples (5 mg) to assess weight loss variations as a result of temperature changes (using TGA-50 Shimadzu). This procedure was carried out at temperatures ranging from 50 to 800 °C, with a heating rate of 10 °C min^−1^ and a nitrogen flow rate of 20 ml min^−1^. This technique is used to test the prepared MOFs' thermal stability.

##### Surface area Brunauer, Emmett and Teller (BET)

The physical adsorption of an N_2_ gas on the surface of the solid and the amount of adsorbate gas corresponding to a monomolecular layer on the surface are used to calculate the specific surface area of the prepared MOFs. Physical adsorption is caused by relatively modest interactions (van der Waals forces) between adsorbate gas molecules and the test samples' adsorbent surface area. A volumetric or continuous flow approach (Quantachrome Corporation Nova 1000, version 6.11 high speed, gas sorption analyzer) at 77 K can be used to determine the amount of gas adsorbed. Before the measurements, the samples were activated at 120 °C for 6 h^21,25^.

##### Optical properties

The samples' optical characteristics were recorded to figure out where in the electromagnetic spectrum they absorb light. The UV–Vis spectrum of prepared MIL-125 was recorded using a UV–Vis spectrophotometer (T60 UV–visible) at wavelength scan ranges from 270 to 650 nm.

#### Photocatalytic degradation test

The prepared MOFs catalysts (MT18, MT48) were tested for the photocatalytic degradation of oil spills using mixed light sources (UV, and visible), and the experimental conditions are shown in Table S2. Each experiment was designed by adding (40 µL) crude oil to (100 mL) seawater under continuous stirring^26^. Firstly, the solution was kept in the dark for 30 min under continuous stirring then irradiation with UV–Vis light (a tungsten lamp of 100-W intensity as a source of visible light and four UV lamps of 2-W each as a source of UV light) was performed and the samples were taken each 30 min and then centrifuged to remove the catalysts. Samples were mixed with an equal volume of toluene with vigorous stirring for 30 min. Leave samples in a separating funnel then measure the remaining hydrocarbons in an aqueous layer by using UV-spectrophotometer at λ = 420 nm and calculate removal efficiency (RE%) using the following equation^27,28^

$$RE\%=\frac{\left[TH\, Control-TH\, treated\right]}{TH\,Control}\times 100$$

where *TH Control* is the oil concentration of the control and *TH Treated* is the oil concentration of the degraded sample.

All the photodegradation experiments were carried out in duplicate with the prepared photocatalyst. Gas Chromatography/Mass Spectrometry (GC/MS Shimazu QP2010 ultra) can help to make conclusive identification of compounds. These were the GC/MS operating conditions; the oven initial temperature was 80 °C for 0 min, raged at 10 °C min^−1^ to 240 °C, and then held for 2 min. The temperature was then ramped to 280 °C at 5 °C min^−1^, and then held at 280 °C for 10 min. The injection temperature and volume were 250 °C and 1 µL, respectively. The split ratio was 20:1. The carrier gas used was He while the solvent delay time was 2.50 min. The transfer and source temperatures were 250 °C and 150 °C, respectively. Scanning was done from 50 to 500 Da and the column dimension was 27.0 m × 250 µm.

## Results and discussion

The reaction of titanium isopropoxide as a metal precursor with terephthalic acid as an organic ligand in a proper percentage of solvent mixtures (*N*,*N*-dimethylformamide (DMF), and methanol) led to well-crystallized white powder of MIL-125. MIL-125 is composed of basic units of Ti_8_O_8_(OH)_4_-(O_2_C-C_6_H_5_-CO_2_)_6_ and is built up from cyclic octamers constructed from corner or edge-sharing octahedral titanium units, as shown in Fig. S1. These octamers are connected to 12 other cyclic octamers through H_2_BDC linkers, leading to a porous three-dimensional quasi-cubic tetragonal structure having two types of cages, an octahedral (12.5 Å) and a tetrahedral (6 Å) cage, accessible through narrow triangular windows of ca. 6 Å^25^.

**Figure 2:**
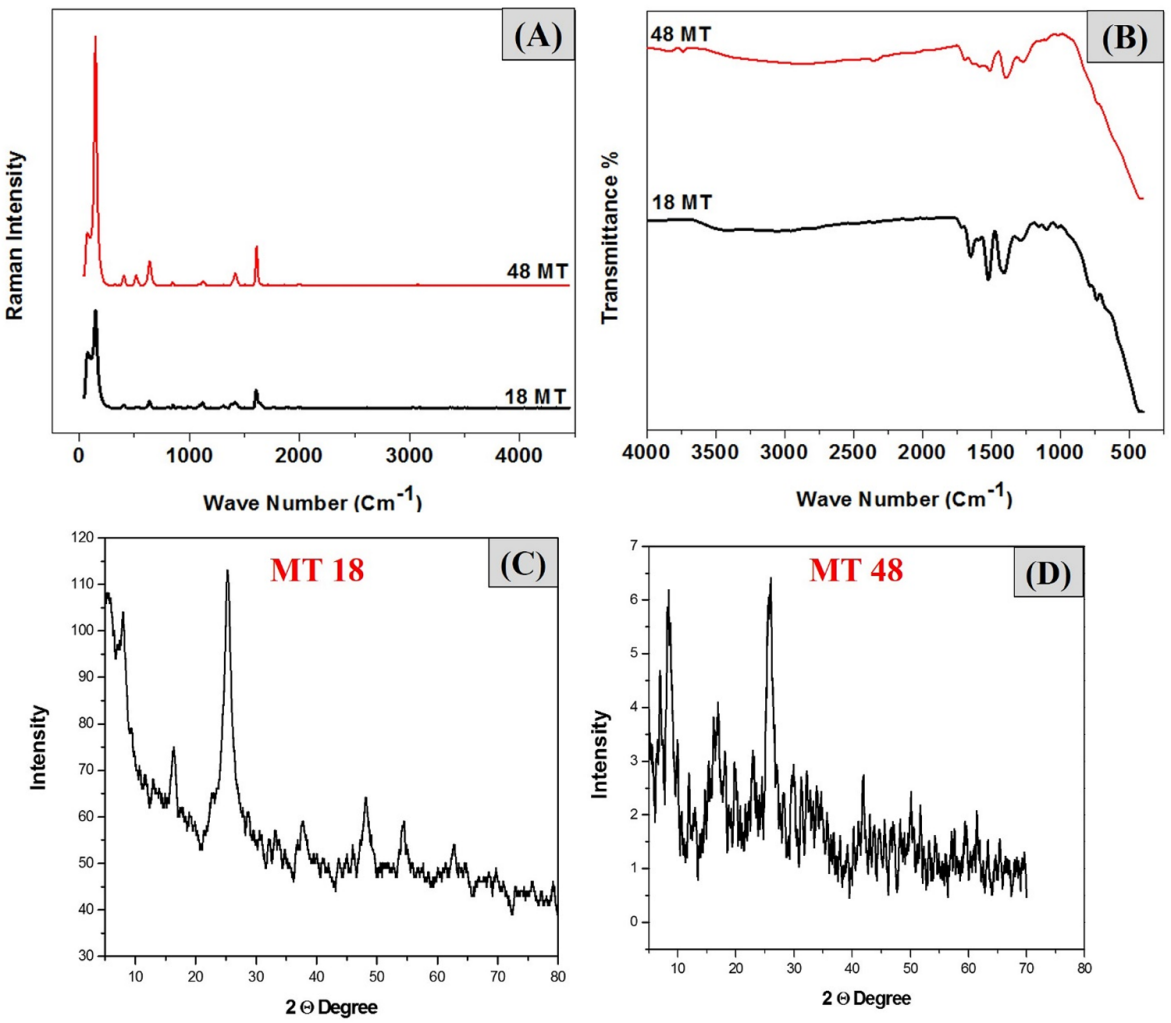
Characterization schemes of the prepared samples (**A**): Raman analysis, (**B**): FTIR analysis, (**C**,**D**): XRD of MT18, and MT48, respectively.

IR and Raman spectra in Fig. 2A,B were used to analyze the structure and morphology of the prepared samples (MT18, MT48). All IR spectra show characteristic vibrational bands in the region of (1400–1700) cm^−1^ for the carboxylic acid functional group of the Ti-coordinated MOF structure. Two absorption bands around (1640, and 1500) cm^−1^ can be assigned to carbonyl asymmetric stretching vibrations, whereas the bands at (1571, and 1628) cm^−1^ could be ascribed to the phenyl C=C ring stretching and the C=O stretching^27^. The band at 1260 cm^−1^ belongs to the C–H symmetric stretching vibrations of the benzene ring, which is assumed by the Raman band at 1611 cm^−1^^20^. The IR region of (400–800) cm^−1^ shows the Ti–O–Ti–O (Ti-oxo clusters) vibrations^20,21^. The bending and symmetric stretching of the framework Ti–O–Ti–O octatomic ring species may be seen at Raman bands (546, 1234, and 1414) cm^−1^. The resonance-enhanced Raman band at 703 cm^−1^ is attributed to the framework titanium species in octahedral coordination settings, i.e., every single titanium atom is coupled to six oxygen atoms. The terephthalic acid ligand activates MT Raman, as evidenced by bands indexed at (1618, 1455, 1181, and 1138) cm^−1^, which correspond to the in-plane vibrational modes of aromatic rings, and bands centered (865 and 641) cm^−1^, which correspond to the vibrational modes of the aromatic rings' C–H and C=C bonds^29^.

XRD displays in Fig. 2C,D, where the porous crystalline carboxylate-based MIL-125 is reported in 2009 by Serre et al., which were synthesized by direct solvothermal^30^. Fine powders of MIL-125 were acquired and their structure was determined by synchrotron powder XRD. The MIL-125 framework, Fig. S1, is composed of cyclic Ti_8_O_8_(OH)_4_(COO)_12_ octamers linked by terephthalic linkers to an edge-and-corner-sharing TiO_5_(OH) octahedron. The units are arranged in a centered cubic (cc) fashion, packing into the quasi-cubic tetragonal lattice, and each octamer has 12 units neighbors. The 3D structure has two types of cages corresponding to the octahedral and tetrahedral vacancies of cc packing, with effectively accessible diameters of 12.55 Å and 6.13 Å, respectively, and connected by triangular windows of 5–7 Å^18^. Because the photocatalytic activity is dependent on the active sites which are also influenced by the crystallite size, which is calculated using the Debye–Scherrer equation. It is found that the crystallite size of MT48 is 7 nm which is a little smaller than for MT18 as shown in Table 1.

**Table 1:** The crystal size calculation from XRD structure according to Debye Scherrer equation.

Samples	FWHM (degree)	2θ (degree)	D (crystal size) (nm)
MT18	1.15	25.19	7.3
MT48	1.14	26.27	7.0

**Figure 3:**
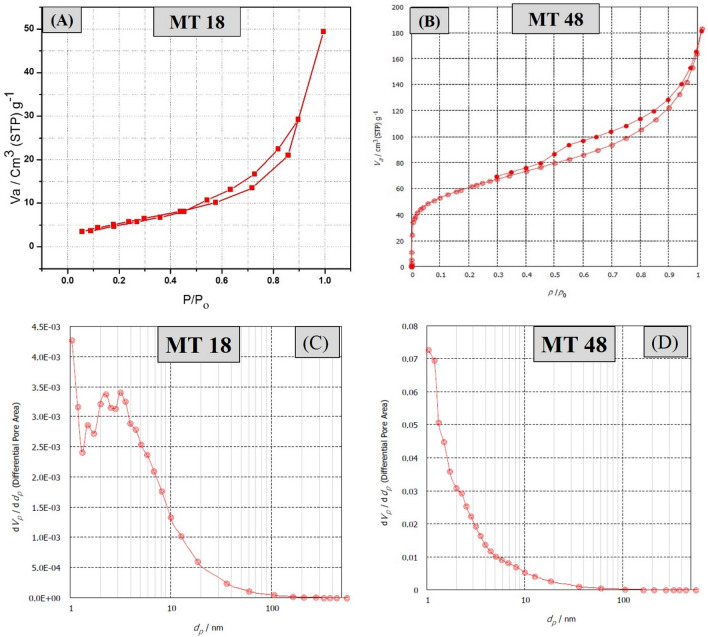
(**A**,**B**) Adsorption/desorption isotherm of both MT18, and MT48 samples, and (**C**,**D**) BJH-plot adsorption branch, adsorptive N2, adsorption temperature 77.350 K of both MT18, and MT48.

The nitrogen adsorption–desorption isotherms, pore diameter, and pore volume of MIL-125 catalysts are shown in Fig. 3. Table 2 shows the pore size distribution displays pores with a diameter between (4.6 and 9.2) nm, for MT48 and MT18 respectively, which correspond to the microporous and mesoporous contributions, respectively, confirming the multimodal distribution. The MT48 catalyst's BET-specific surface area and total pore volume were 301 m^2^ g^−1^ and 0.2444 cm^3^ g^−1^ respectively, showing that it is a highly porous material. More active sites would be exposed if mesopores with a bigger surface area were formed, which would boost photocatalytic activity.

**Table 2:** Textural properties of MIL-125 catalysts.

Samples	S_BET_ (sq. m g^−1^)	Total pore volume (V pore) (cm^3^ g^-1^)	Average pore diameter (nm)
MT18	21.07	0.040083	9.2604
MT48	301.10	0.2444	4.6352

**Figure 4:**
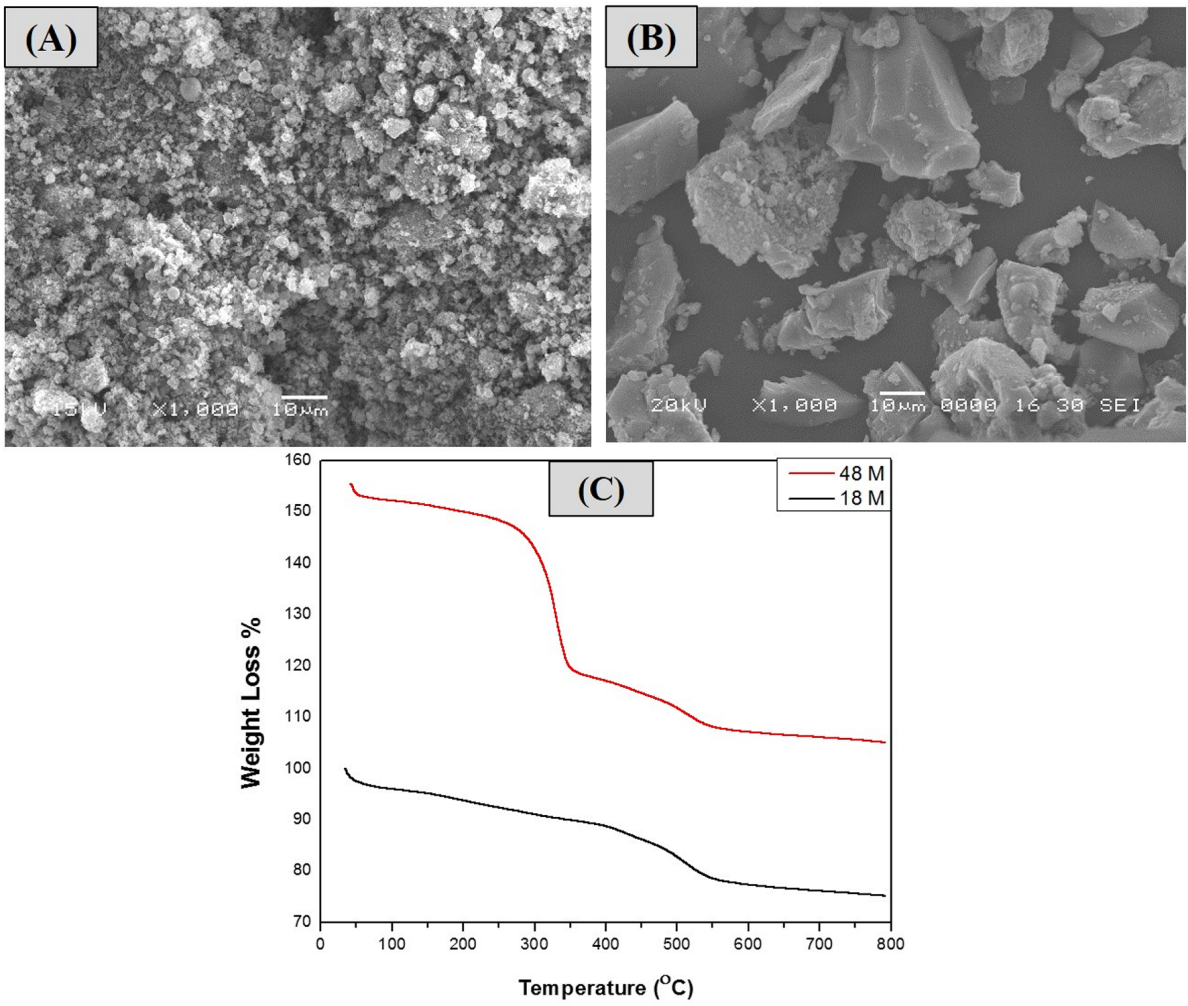
SEM images of (**A** MT18, **B** MT48), and (**C**) thermogravimetric analysis (TGA) of both MT18, MT48.

SEM images Fig. 4A,B reveal that both (MT18, MT48) samples are made up of particles with typical circular plate-like morphology^9^ with particle sizes (258 ± 89, 234 ± 65) nm, respectively. The thermogravimetric analysis (TGA), in Fig. 4C, indicates a steady mass loss of the prepared MOFs before 800 °C. The first weight loss before 200 °C at (80 °C, 61 °C) with weight loss (3.6%, 2.6%) for (MT18, MT48), respectively. This may be to the elimination of physically adsorbed gases, moisture, and DMF from the pores presumably owing to solvate evaporation^31^. The second weight loss between (200–500) °C is due to the framework disintegration^32^, which eventually produces amorphous TiO_2_ residue^33^. The residues were (75.33, and 49) % of the initial mass for both (MT18, and MT48), respectively.

### Optical characterization

**Figure 5:**
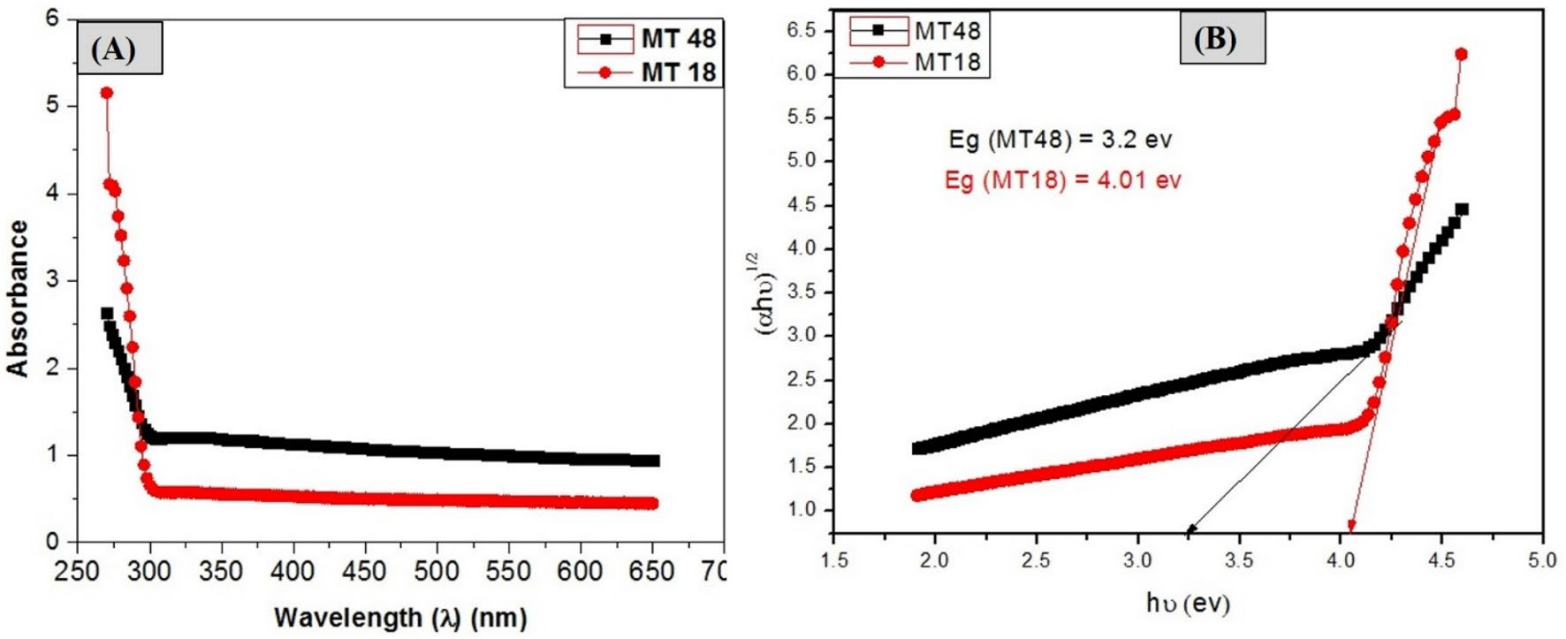
Optical behavior of MT18, MT48 (**A**) UV–Vis spectrum, (**B**) optical band gap plot.

The optical properties of prepared MOFs (MT18, MT48) were investigated using the UV–VIS spectrum which is shown in Fig. 5A. Due to the octahedral coordination in titanium species, the two prepared samples (MT18, MT48) possess a broad absorption edge at wave length (310 nm), MT18, MT48 absorb only at wavelength below 310 nm, inducing excitation from HOMO, made of π states in the ligand and O(2p) in the metal-oxo cluster, into the LUMO defined by Ti(3d), leaving behind holes near the Ti-oxo cluster^9,34,35^. The optical bandgap (Eg) demonstrates a relationship between the absorption coefficient and the incident photon energy and can be calculated by using the following Tauc’s equation^35,36^.

$$\alpha h\nu=A\left(h\nu-Eg\right){\text{n}}$$

$$h\nu=1240/\lambda$$

where α is the coefficient of the absorption, hυ is the incident photon energy, A is a constant, Eg represents the bandgap, and n is determined based on the type of optical transition of prepared MOFs.

These materials have an indirect optical transition, thus n = 2. The optical energy gap (Eg) can be deduced from plotting the relationship between (αhʋ)^1/2^ and photon energy (hʋ) for MT18 and MT48 as shown in Fig. 5B. The calculated Eg of MT18 and MT48 is ∼ 4.03 eV and ∼ 3.2 eV, respectively, from the intercept of the tangent to the line. The reduction of band gap values from 4.03 eV for MT18 to 3.2 eV for MT48 may be due to the particle size decreasing from (258 to 234) nm as the solvothermal reaction time increase from (18 to 48) h^37^.

### Photocatalytic degradation of crude oil spills

**Figure 6:**
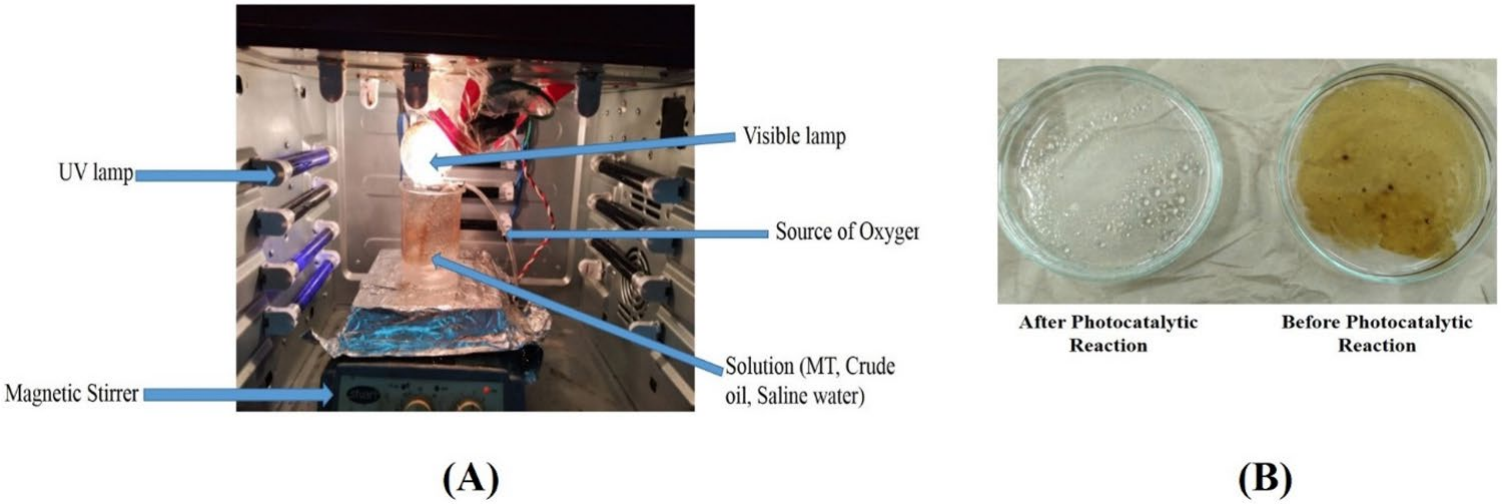
Photo-degradation study of (crude oil/saline water) samples by using different prepared MOF samples (18 MT, 48 MT).

**Figure 7:**
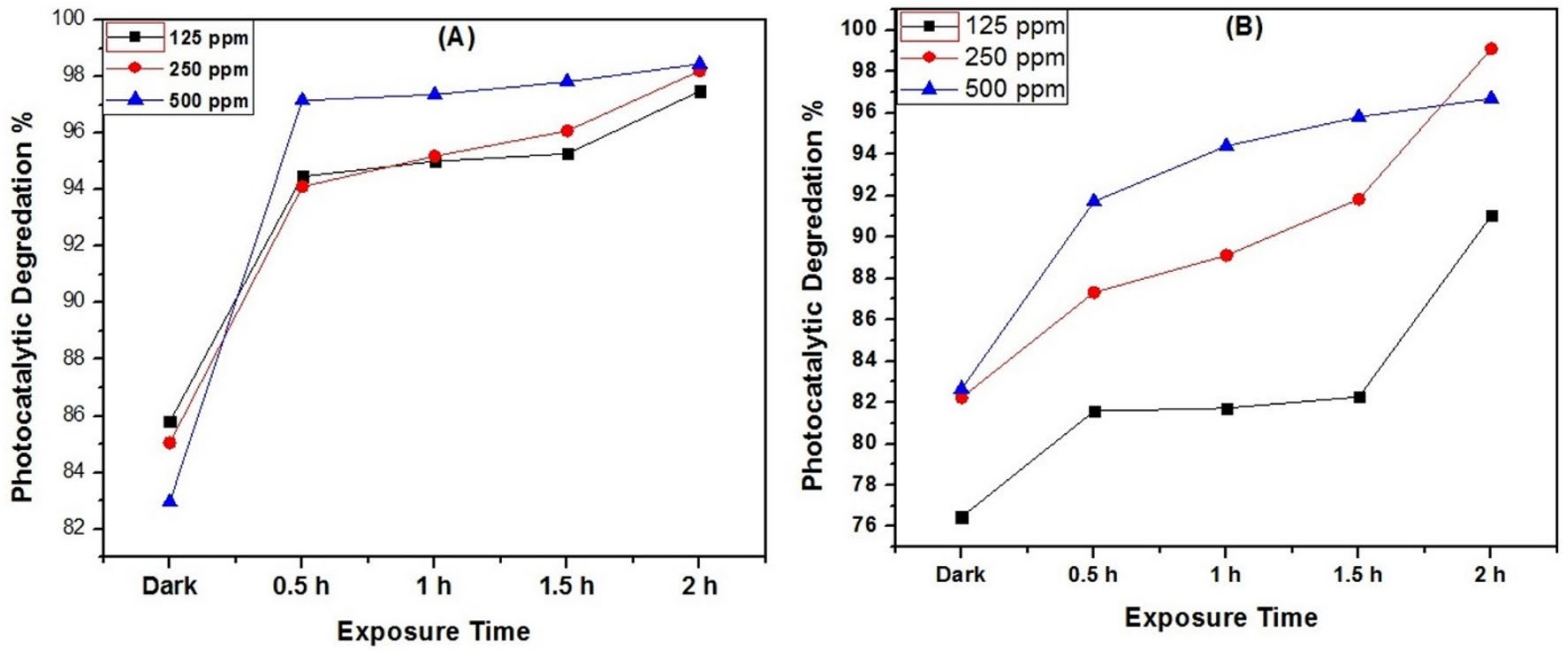
Photocatalytic degradation efficiency of crude oil, using different prepared samples (**A** MT18 and **B** MT48).

Figure 6 shows the photo-degradation study of (crude oil/saline water) samples by using different MOF catalysts and it clearly shows that the disappearance of oil spills after photocatalytic reaction using MT18 and MT48. The concentration of crude oil before and after photodegradation can be determined using UV–Vis spectrophotometry and GC/MS technique. Figure 5 shows the UV–Vis spectrophotometry of degraded crude oil with respect to using the light as controlled value (absorbance = 3.344). Generally, the two prepared MIL-125 samples display a high efficiency toward the degradation of crude oil. Figure 7A shows that the use of different doses of MT18 as photocatalyst (125, 250, and 500) ppm in the degradation of crude oil spill revealed high degradation efficiencies reached 98.4% after 2 h of irradiation. On the other hand, Fig. 7B shows that using 250 ppm of MT48 as photocatalyst can degrade about 99% of crude oil spills after 2 h of irradiation. The MT48 has little more degradation efficiency under (UV–Vis light) this is due to the lower optical band gap (3.2 eV) and higher surface area (301.1 m^2^ g^−1^)^37^. The trend observed in the UV–Vis spectrophotometry analysis is agreed with the optical properties and morphological characterization results of prepared photocatalyst.

**Figure 8:**
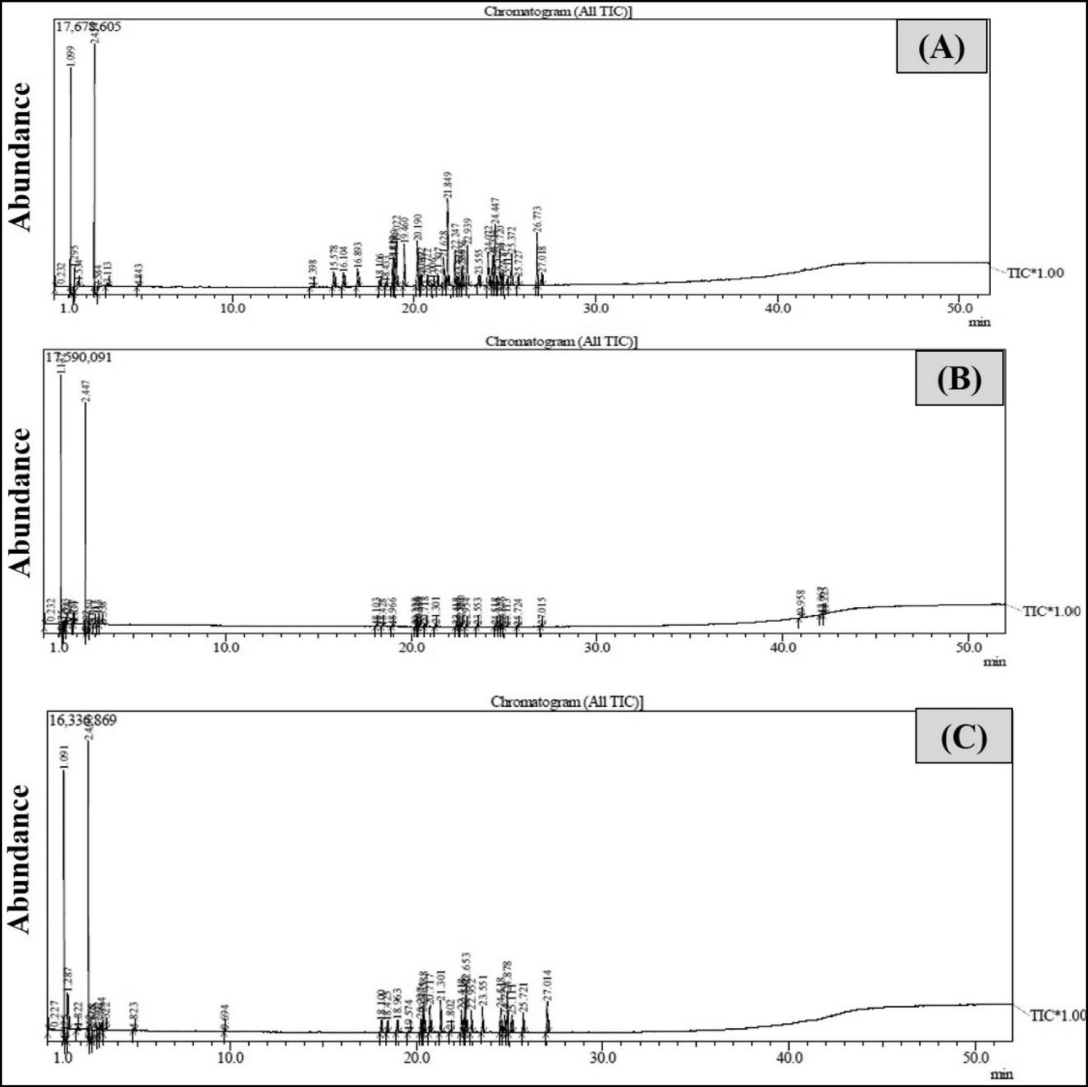
Chromatogram of crude oil photocatalytic degradation (**A** control, **B** degraded oil using MT18, and **C** degraded oil using MT48) after 2 h irradiation.

The GC/MS chromatograms of the analyzing wastewater samples before and after photodegradation are presented in Fig. 8. This analysis shows that water contaminated with crude oil spills (Control) contains different compounds such as Pentane-2-methyl, Tetrahydrofuran (THF), Cyclohexane, 6-Bromo-4-(2-choro-phenyl)-2-piperan, Benzene, (1-butylhexadecl), Benzene, (1-methylnonyl), Benzene, (1-buylheptyl), Benzene, (1-propyloctyl), Octadecane and Benzene, (1-pentylheptyl). The obtained chromatograms after photocatalytic degradation using MT18 and MT48 cleared that all mentioned compounds are degraded with high efficiencies and their concentrations become nil^38^.

### Kinetics of the reaction rate

**Figure 9:**
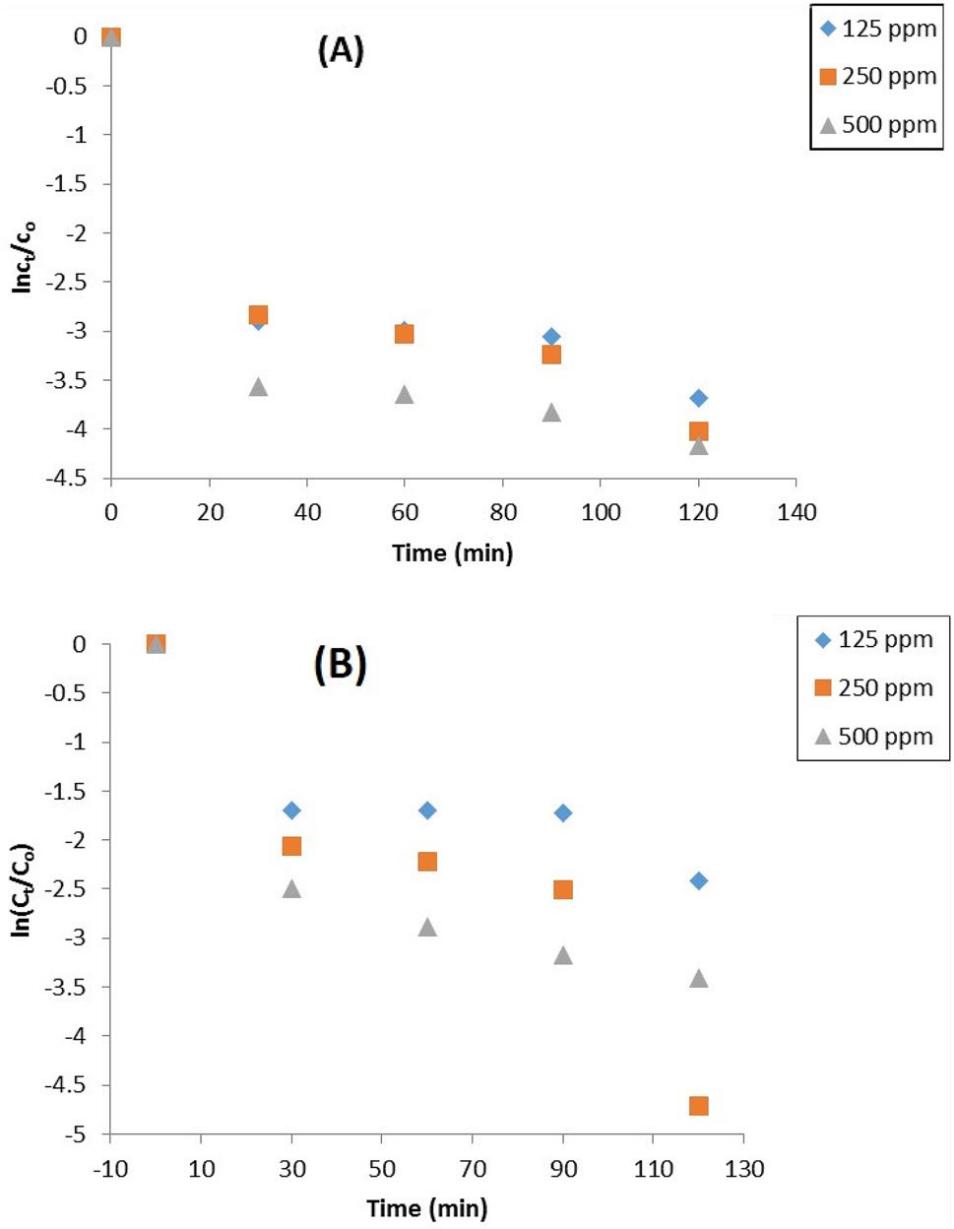
Kinetic study of photocatalytic degradation of oil spills using (**A**) MT18, (**B**) MT48.

Under various experimental conditions, our findings indicated that MIL-125 could be used to degrade marine oil spills. The photoreaction process can be divided into two stages based on the kinetic reaction curves in Fig. 9. First, the concentration decreased rapidly at the start of the irradiation, and the photoreaction confirmed a first-order kinetic pattern. Secondly, the concentration was nearly stabilized. According to the first-order kinetic reaction:

$$ln(Co/Ct)=kt$$

$$t1/2=ln2/k$$

where *C*_*o*_ is the initial reaction concentration of oil; *C*_*t*_ is the concentration of oil measured at a fixed time; t is irradiation time, and $K$ is is the reaction rate determined by the linear plot of In (*C*_*o*_/*C*_*t*_) versus the time. Table 3 summarizes the kinetic data from this study's photo-degradation of oil under various experimental conditions. According to our results, photochemical degradation is an effective method for removing petroleum hydrocarbons from seawater.

**Table 3:** Kinetic data of photodegradation of oil spills using prepared MOFs under different conditions.

Catalyst dose (ppm)		k (min^-1^)		t_1/2_ (min)	
MT18	MT48	MT18	MT48	MT18	MT48
125	125	0.025	0.0162	4.382	4.816
250	250	0.028	0.0329	4.268	4.107
500	500	0.0286	0.025	4.247	4.382

## Conclusions

From this research, it is possible to conclude that, efficient MIL-125 photocatalysts have been successfully prepared via a facile solvothermal method. The sample prepared at 150 °C and a reaction time of 48 h (MT48) has a low crystal size of 7 nm and a particle size of 234 nm with an optical bandgap of 3.2 eV and a surface area of 301 m^2^ g^−1^. Based on GC/MS and UV/Vis photodegradation results, the two prepared samples show high efficiency toward the degradation of crude oil. And the use of 250 ppm of MT48 photocatalyst under UV–Vis irradiation can degrade about 99% of oil spills in water. This study presents a novel and highly efficient photocatalytic degradation of crude oil spills using MIL-125.

## Supplementary Information

Supplementary Information.

## Data Availability

The generated and analyzed data during the current study supplied in this manuscript and is readily available from the corresponding author upon reasonable request.
