# Publisher Correction: Efficient photocatalytic degradation of petroleum oil spills in seawater using a metal-organic framework (MOF)

**DOI:** 10.1038/s41598-023-30138-5

**Published:** 2023-02-20

**Authors:** M. S. Showman, Asmaa M. Abd El‑Aziz, Rana Yahya

**Affiliations:** 1 Fabrication Technology Research Department, Advanced Technology and New Materials Research Institute, City of Scientific Research and Technological Applications, Alexandria, Egypt; 2 Department of Chemistry, College of Science, University of Jeddah, Jeddah, Saudi Arabia

Correction to: *Scientific Reports* 10.1038/s41598-022-26295-8, published online 23 December 2022

The original version of this Article contained an error in Affiliation 2, which was incorrectly given as ‘Department of Chemistry, College of Science, University of Jeddah, Jidda, Saudi Arabia’.

The correct affiliation is listed below:

Department of Chemistry, College of Science, University of Jeddah, Jeddah, Saudi Arabia

The original Article has been corrected.

