# Supplementary material for: Efficient photocatalytic degradation of petroleum oil spills in seawater using a metal-organic framework (MOF)
Source: Sci Rep. 2022 Dec 23;12:22445. doi: 10.1038/s41598-022-26295-8 (PMC9794805; doi:10.1038/s41598-022-26295-8)
Supplement: Supplementary file 1 — Supplementary Information. [file 41598_2022_26295_MOESM1_ESM.pdf]

Supplementary Data

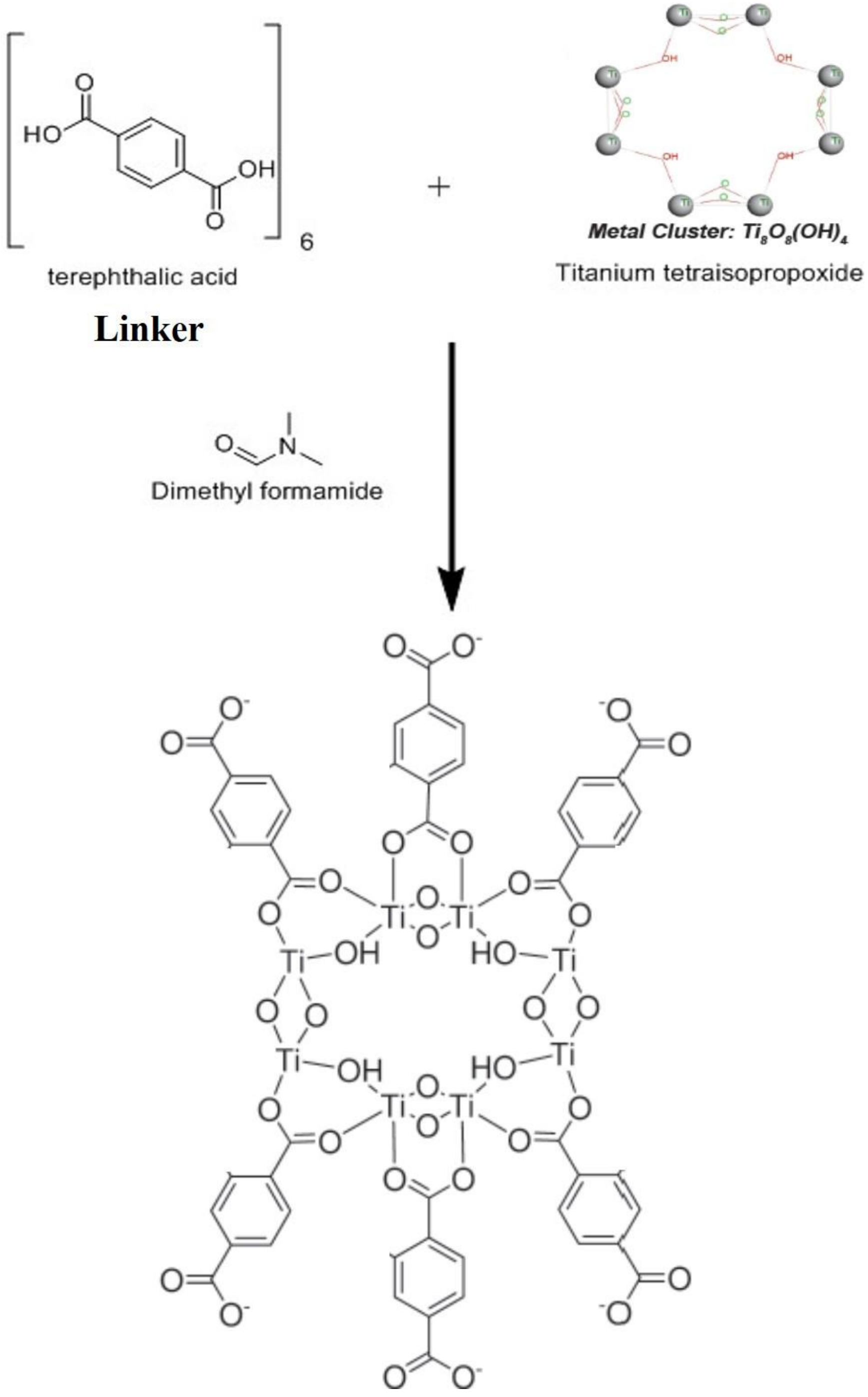

Figure S1: Mechanism of the reaction to form the Ti-MOF catalyst.

Reaction of Ti(iOPr)4 and the organic ligand, terephthalic acid, in an appropriate proportion of solvent mixtures (N,N0-dimethylformamide (DMF) and methanol) at 423 K for 15 h successfully led to well crystallized white powder of MIL-125.

As shown in S1, MIL-125 is composed of basic units of Ti8O8(OH)4-(O2C- C6H5-CO2)6, and is built up from cyclic octamers constructed from corner or edge sharing octahedral titanium units. These octamers are connected to 12 other cyclic octamers through BDC linkers, leading to a porous three-dimensional quasi-cubic tetragonal structure having two types of cages, an octahedral (12.5 ˚A) and atetrahedral (6 ˚A) cage, accessible through narrow triangular windows of ca. 6 ˚A (Kim et al., 2013)

Table (S1): Western desert crude oil main characteristics.

Test	Result
Density (kg /cm3)	0.8156
API Gravity	41.99
pour point	+ 3
Sulfur (mass %)	0.36
Salt content (vol %)	0.0018
Redwood Viscosity at 100 F (sec)	39
Conradson Carbon (Wt %)	2.12

Table (S2): Suggested experimental conditions of the photo-degradation experiments.

Test Number	Conditions
1	Light without MOF catalyst
2	Dark with MOF Catalyst
3	Light with different dose of the catalyst (withdraw sample after four time intervals) (light + MOF).
